# Enhanced Susceptibility to Tomato Chlorosis Virus (ToCV) in *Hsp90-* and *Sgt1*-Silenced Plants: Insights from Gene Expression Dynamics

**DOI:** 10.3390/v15122370

**Published:** 2023-11-30

**Authors:** Irene Ontiveros, Noé Fernández-Pozo, Anna Esteve-Codina, Juan José López-Moya, Juan Antonio Díaz-Pendón

**Affiliations:** 1 Institute for Mediterranean and Subtropical Horticulture La Mayora (IHSM), CSIC-UMA, 29750 Algarrobo-Costa, Spain; irene.ontiveros@csic.es (I.O.); noe.fernandez.pozo@csic.es (N.F.-P.); 2 Centre for Research in Agricultural Genomics (CRAG), CSIC-IRTA-UAB-UB, 08913 Bellaterra, Spain; 3 CNAG-CRG, Centre for Genomic Regulation (CRG), Barcelona Institute of Science and Technology (BIST), 08028 Barcelona, Spain; anna.esteve@cnag.eu

**Keywords:** basal resistance, *Hsp90*, *Sgt1*, ToCV, *Bemisia tabaci*, tomato

## Abstract

The emerging whitefly-transmitted crinivirus tomato chlorosis virus (ToCV) causes substantial economic losses by inducing yellow leaf disorder in tomato crops. This study explores potential resistance mechanisms by examining early-stage molecular responses to ToCV. A time-course transcriptome analysis compared naïve, mock, and ToCV-infected plants at 2, 7, and 14 days post-infection (dpi). Gene expression changes were most notable at 2 and 14 dpi, likely corresponding to whitefly feeding and viral infection. Gene Ontology (GO) and Kyoto Encyclopedia of Genes and Genomes (KEGG) enrichment analyses revealed key genes and pathways associated with ToCV infection, including those related to plant immunity, flavonoid and steroid biosynthesis, photosynthesis, and hormone signaling. Additionally, virus-derived small interfering RNAs (vsRNAs) originating from ToCV predominantly came from RNA2 and were 22 nucleotides in length. Furthermore, two genes involved in plant immunity, *Hsp90* (heat shock protein 90) and its co-chaperone *Sgt1* (suppressor of the G2 allele of Skp1) were targeted through viral-induced gene silencing (VIGS), showing a potential contribution to basal resistance against viral infections since their reduction correlated with increased ToCV accumulation. This study provides insights into tomato plant responses to ToCV, with potential implications for developing effective disease control strategies.

## 1. Introduction

Tomato chlorosis virus (ToCV, genus *Crinivirus*, family *Closteroviridae*) is an emergent plant virus that causes a yellow leaf disorder in tomatoes, including interveinal yellowing chlorotic areas, thickening of leaves, and bronzing and necrosis of the older leaves, which may cause serious economic losses in crop yield [1,2]. ToCV has a bipartite genome of positive single-stranded RNA and, like many other criniviruses, is restricted to phloem-associated cells and transmitted in a semi-persistent manner by several whiteflies of the genera *Bemisia* and *Trialeurodes*, although its emergence has been associated mostly with the global spread of the whitefly *B. tabaci* in tropical and warm regions worldwide [1]. Furthermore, recent studies have demonstrated that ToCV, when present in a mixed infection with tomato yellow leaf curl virus (TYLCV, genus *Begomovirus*, family *Geminiviridae*), also transmitted by *B. tabaci*, might result in a synergistic interaction disease that could have a significantly detrimental impact on tomato production [3,4,5,6].

Since plant viruses are obligate intracellular parasites that exploit host cellular machinery to establish systemic infections [7], they generally induce a wide variety of alterations in host gene expression and cell physiology in order to facilitate infections. These alterations include not only defense-related pathways but also some others involved in photosynthesis, secondary metabolism, or regulation of plant hormone levels [8,9,10,11,12]. Transcriptome sequencing using RNA-seq technology allows the exploration of gene expression changes that are either directly or indirectly associated with viral infection [13,14,15]. Previous studies have followed this approach to characterize genome-wide gene expression profiles in tomatoes in response to ToCV infection [16,17]. These two papers focused on a single time point after inoculation for their sampling and analysis. In the first work, Seo et al. (2018) [16] performed transcriptome analysis of grafted plants from ToCV-infected tomato plants at 56 days post-inoculation (dpi), pointing to genes potentially involved in the response affecting the development. Instead, Yue et al. (2021) [17] analyzed plants infected by agroinoculation with an infectious clone at 40 dpi, identifying in this case genes associated with the MAPK signaling pathway, the glyoxylate cycle, and photosynthesis processes. This variability in the observed responses might reflect experimental differences in terms of virus isolates, plant materials, and especially inoculation modes. Furthermore, it is important to consider the dynamic nature of the pathogenic interaction. In particular, the changes in gene expression in response to viral infection are highly dynamic, and plant viruses are known to induce changes in host gene expression at the early stages of infection, which leads to the activation of antiviral responses [8,18,19]. Therefore, our understanding of how ToCV infection processes regulate gene expression during the early stages of natural vector-mediated infection remains limited.

RNA silencing is a well-established antiviral defense system in plants [20,21]. This antiviral defense involves the production of virus-derived small interfering RNAs (vsRNAs) by RNase III Dicer-like (DCL) proteins processing viral RNA precursors into 21- to 24-nucleotide (nt) RNAs [22,23]. In *Arabidopsis thaliana*, virus resistance against positive-strand RNA viruses is initiated by either DCL4 or DCL2, which are involved in the biogenesis of 21 nt and 22 nt vsRNAs, respectively [24,25,26]. These vsRNAs are loaded onto AGO proteins, leading to the formation of RNA-induced silencing complexes (RISCs) that repress complementary target RNA [27]. Moreover, the 22 nt vsRNAs are likely to promote the amplification of RNA silencing, which includes the production of secondary vsRNAs by DCL proteins from the products of RNA-dependent RNA polymerases (RDRs) [28,29]. Characterization of vsRNAs through deep sequencing techniques in response to various plant viruses has been previously conducted in multiple agronomically important crop species [30,31,32,33,34,35]. Nevertheless, the vsRNA profile originating from ToCV infection in tomato plants has only been documented in a single study, which analyzed the tomato virome through sequencing sRNAs from field crop samples collected in China [35]. Consequently, our understanding of the vsRNA profile associated with ToCV infection in tomato plants remains limited.

In this study, we performed time-course transcriptome analysis using RNA-seq to analyze the dynamic changes of differentially expressed genes (DEGs) in tomato plants after ToCV infection when transmitted by its natural insect vector *B. tabaci*. Key genes were comprehensively identified and classified into essential pathways, providing new insight into ToCV pathogenesis and the host immune response. Further analyses of the distribution of vsRNAs along the viral genome determined using sRNA sequencing indicated that RNA1 and RNA2 were differentially targeted by vsRNAs. We also observed that genes involved in plant immunity, such as *Hsp90* (heat shock protein 90) and its co-chaperone *Sgt1* (suppressor of the G2 allele of Skp1), may contribute to the basal resistance to viral infection. These findings provide new insights into the molecular responses that occur in ToCV-infected tomato plants and may represent a step toward identifying potential genes for designing future disease control strategies against ToCV.

## 2. Materials and Methods

### 2.1. Plant Material and Virus Infection

Tomato plants (*Solanum lycopersicum* cv. Moneymaker) were grown in an insect-free growth chamber with a 16 h photoperiod at 250 μmol·s^−1^m^−2^ photosynthetically active radiation (25 °C/20 °C) and 70% relative humidity [36]. The isolate of ToCV used in this study was the Pl-1-2 [37], maintained at IHSM La Mayora, CSIC-UMA in the tomato cv. Moneymaker by periodic transmission with *B. tabaci* Mediterranean (MED). A nonviruliferous colony of *B. tabaci* was reared on melon plants in cages covered by insect-proof netting. To obtain ToCV-viruliferous whiteflies, nonviruliferous whiteflies were exposed for a 24 h acquisition access period (AAP) on tomato plants infected with ToCV at the 7–8 true-leaf stage that were inoculated four weeks before being used for virus acquisition, as previously described [6]. Similarly, whiteflies used as non-viruliferous controls (mock) were enclosed in clip cages attached to virus-free tomato plants for the same 24 h period. After the AAP, 40 whiteflies per test plant in clip-on cages were transferred to the 3rd true leaf of healthy tomato plants for a 48 h inoculation access period (IAP). Following IAP, the clip cages were removed and the infested leaf was excised from the plant at 7 days to avoid eclosion of eggs laid by adults during the IAP. The plants were maintained in an insect-free growth chamber (see above).

### 2.2. Sample Collection and RNA Extraction

Naïve (no whitefly and no virus), mock (non-viruliferous whiteflies), and ToCV (viruliferous whiteflies)-treated samples were collected at 2, 7, and 14 days post-infection (dpi). The presence of viral RNA in the inoculated plants was tested by tissue blot hybridization 21 dpi as described, and only samples from plants that tested positive were included in the pool [38]. At each time point and replica, the second most recently expanded leaves from the apex of 6 individual plants were pooled and used in downstream analysis. A total of three biological replicates were processed per treatment and time point. Total RNA was extracted using TRI reagent (Invitrogen by Thermo Fisher Scientific, Vilnius, Lithuania) according to the manufacturer’s instructions and then treated with RNase-Free DNase (Qiagen, Hilden, Germany). The quality and quantity of RNA were assessed by electrophoresis on 1% agarose gels and with a NanoDrop 1000 spectrophotometer (Thermo Fisher Scientific, Madison, WI, USA).

### 2.3. Libraries Construction and Sequencing

RNA-Seq libraries were generated and sequenced at CNAG (Centro Nacional de Análisis Genómico, Barcelona, Spain). Total RNA was assayed for quantity and quality using Qubit^®^ RNA HS Assay (Life Technologies, Austin, TX, USA) and RNA 6000 Nano Assay on a Bioanalyzer 2100. The RNASeq libraries were prepared from total RNA using the TruSeq^®^Stranded mRNA LT Sample Prep Kit (Illumina Inc., San Diego, CA, USA Rev.E, October 2013). A total of 27 libraries were constructed (3 for each treatment; 3 biological replicates at 2, 7, and 14 dpi). The libraries were sequenced on HiSeq2000 (Illumina, Inc.) in paired-end mode with a read length of 76 bp using the TruSeq SBS Kit v4. Image analysis, base calling, and quality scoring of the run were processed using the manufacturer’s software Real Time Analysis (RTA 1.18.64 or 1.18.66.3) followed by the generation of FASTQ sequence files by CASAVA.

sRNA libraries were generated and sequenced at CRG (Centre for Genomic Regulation, Barcelona, Spain) as described by [36]. Briefly, libraries were prepared in duplicate from samples corresponding to ToCV-infected plants at 14 dpi using the Illumina TruSeq small RNA sample prep kit according to the manufacturer’s instructions. Libraries were validated on an Agilent 2100 Bioanalyzer (Agilent Technologies, Santa Clara, CA, USA) using a DNA High Sensitivity chip and quantified by qPCR using the Kapa Library Quantification kit for Illumina (Roche, Basel, Switzerland). Sequencing was performed on an Illumina HiSeq2500 using 50 bp single reads with HiSeq v4 sequencing chemistry.

### 2.4. RNA-Seq and sRNA Data Analysis

RNA-seq raw data files obtained after sequencing were processed using Trimmomatic v0.35 [39] with the options ILLUMINACLIP:./TruSeq3-PE.fa:2:30:10 LEADING:3 TRAILING:3 SLIDINGWINDOW:4:15 MINLEN:36 to remove sequencing adapters and low-quality reads. Raw and processed reads were evaluated with FastQC v0.11.4 (https://www.bioinformatics.babraham.ac.uk/projects/fastqc/, accessed on 7 March 2023) and MultiQC v1.6. [40]. Processed reads were mapped to the current version of the tomato reference genome (SL4.0) available at the Sol Genomics Network website (SGN, http://solgenomics.net, accessed on 9 March 2023) [41], together with the tomato organelle sequences, using Hisat v2.1.0 [42]. Samtools v1.9 [43] was used to convert the alignment to a sorted BAM format. StringTie v1.3.3b [44] was used for transcript quantification based on the tomato ITAG4.1 annotation. The Python script prepDE.py was used to convert Stringtie output to counts, and differentially expressed genes (DEGs) were calculated using the R package DESeq2 [45], applying a cutoff threshold of 1.2-fold compared to the values observed in both the ToCV and mock treatments. Gene set enrichment analyses of the DEGs were performed with g:profiler [46] (https://biit.cs.ut.ee/gprofiler/gost, accessed on 28 April 2023). To determine the similarity across samples, rlog-transformed data were used for principal component analysis (PCA) using plotPCA of DEseq2 and pairwise comparison of all samples using Pearson’s correlation coefficients in R.

sRNA raw data files obtained after sequencing were processed using Trimmomatic v0.35 [39] with the options ILLUMINACLIP:./smrna_adapters.fa:2:30:10 LEADING:3 TRAILING:3 SLIDINGWINDOW:4:15 MINLEN:15 to remove specific smallRNA adapters and low-quality reads. Then, trimmed reads <18 nt or >30 nt were removed. Raw and processed reads were evaluated with FastQC v0.11.4 (https://www.bioinformatics.babraham.ac.uk/projects/fastqc/, accessed on 23 May 2023) and MultiQC v1.6 [40]. Processed reads were aligned using BWA v0.7.12 [47], with the edit distance set to 1, to the genome of RNA1 (GenBank accession number KJ200308) and RNA2 (GenBank accession number KJ200309) of ToCV. The sRNA size distribution of 20 to 25 nt, total read counts, and the counts for forward and reverse orientation were estimated using MISIS2 [48].

### 2.5. Virus-Induced Gene Silencing

VIGS vectors (pTRV1 and pTRV2) derived from the tobacco rattle virus (TRV) [49] were used to silence *Sgt1* (Solyc06g036420) and *Hsp90* (Solyc07g047790; contains interpro domain(s) IPR001404 Heat shock protein Hsp90) genes. Gene-specific PCR primer pairs were designed using the Primer Blast tool available online (https://www.ncbi.nlm.nih.gov/tools/primer-blast/, accessed on 10 June 2021) and fragments were designed by the VIGS tool (http://solgenomics.net/tools/vigs, accessed on 10 June 2021) [41]. A 430 bp fragment of the *Hsp90* gene was amplified by RT-PCR from total RNA (see below). The primers used were LK35A (5′-GATCGGATCCTTGAGCAGTTCTCCTTGTGT-3′) and LK36A (5′-AGTCGAGCTCATTTCTGTCCACCAGCTTCA-3′), which contained BamHI and SacI restriction enzyme sites, respectively, as indicated by underlining in the sequence. The resulting PCR product was digested and then cloned into the BamHI-SacI sites within the multiple cloning region of the pTRV2 vector. Similarly, a 235 bp fragment of *Sgt1* was amplified using the primers LK22A (5′-TAGTGAATTCATCCTGCATCTGAGTTACCG-3′) and LK23A (5′-GCATCTCGAGGTTTCTTCACCTGGCACATC-3′) and inserted into EcoRI-XhoI sites of the pTRV2 vector after restriction digestion using the corresponding EcoRI and XhoI restriction enzyme sites underlined in the sequence of primers. Recombinant plasmids were transformed into *Escherichia coli* strain DH5α (Invitrogen) and the corresponding plasmids of clones pTRV2-*Hsp90* and pTRV2-*Sgt1* were extracted and electroporated into *Agrobacterium tumefaciens* GV3101. Bacterial cultures were grown at 28 °C for 48 h in liquid LB medium containing kanamycin (50 µg/mL) and rifampicin (25 µg/mL), and then harvested by centrifugation and the pellets were resuspended in infiltration buffer (10 mM MgCl_2_, 10 mM MES pH5.6, 200 μM acetosyringone) to a final OD600 of 1.0. Agrobacterium cultures containing pTRV2 derivatives and pTRV1 were mixed in a 1:1 ratio, incubated at room temperature for 3 h, and then infiltrated into a 2–3-leaf-stage tomato. The pTRV2 empty vector was used as the control together with pTRV1. Seven days after inoculation with the different TRV-based constructs, plants were infected with ToCV by *B.tabaci*-mediated inoculation as described above. The second most recently expanded leaves from the apex were harvested from 5 plants in each treatment and used in downstream analysis.

### 2.6. Quantitative Real-Time PCR

RNA isolation from plant samples was performed using TRIZOL following standard protocols. Reverse transcription and quantitative PCR (RT-qPCR) were carried out as previously described [6,50], with some slight modifications. Specifically, each treatment included five biological replicates and three technical replicates, and we used the elongation factor 1-α and Sand as reference genes [51,52].

Quantification of ToCV and TRV accumulation was carried out as described in [52]. Specific primer pairs for qPCR LK57 (5′-GGTGTTACTGAGCCTGAGC-3′) and Lk58 (5′-GGCGAGTCATACCAATTCCTG-3′) were used to amplify a 115 nt segment of the *Hsp90* gene, and LK53 (5′-CCAAGATGCTGACGAGGAC-3′) and LK54 (5′-CAGAGGATCGATTCTAGATCTCC-3′) to amplify a 166 nt segment of the *Sgt1* gene. Each primer pair was evaluated using a standard curve with six points and three replicates to obtain efficiency rates (E) of 104.73% (for *Hsp90*) and 101.74% (for *Sgt1*) (E = 10(1/slope) − 1, expressed as percentages) with R2 = 0.99 correlation values for the curves. Relative gene expression was calculated using the 2^−ΔΔCT^ method [53]. Statistically significant differences (*p* < 0.05) in virus accumulation and target gene expression levels in control and silenced tomato plants were analyzed using one-way ANOVA.

## 3. Results

### 3.1. Identification of DEGs in Tomato Leaves at Different Stages of ToCV Infection

**Figure 1:**
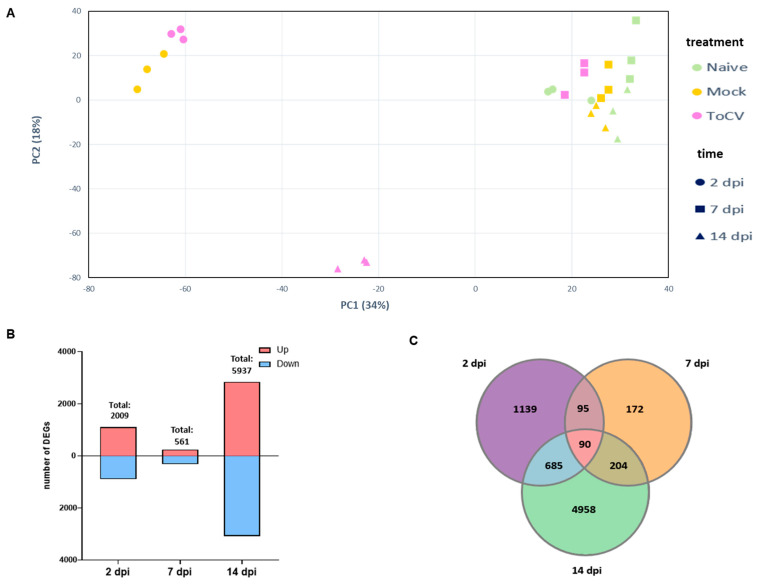
Exploratory analysis of transcriptomic data. (**A**) Principal component analysis (PCA) of all replicates from naïve (no whitefly and no virus), Mock (non-viruliferous whiteflies), and ToCV (ToCV-viruliferous whiteflies) samples at 2, 7, and 14 days post-infection (dpi). (**B**) Number of up-regulated and down-regulated differentially expressed genes (DEGs) at each time point after ToCV infection. Red and blue bars represent numbers of up-regulated and down-regulated genes, respectively. Total indicates the total number of both up-regulated and down-regulated DEGs. (**C**) Venn diagram displaying the number of shared and distinct DEGs across the three specified time points.

To investigate changes in the tomato transcriptome profile associated with ToCV infection, Illumina TruSeq libraries were sequenced from the total RNA of naïve, mock, and ToCV-infected plants at 2, 7, and 14 dpi. The number of clean reads ranged from 27 to 72 million read pairs, and the percentage of mapped reads against the tomato transcriptome ranged from 90.96 and 92.68. PCA analysis showed that the samples clustered into three discrete groups (Figure 1A). A clear discrimination between ToCV-infected and uninfected samples was only found at 14 dpi. Also, a clear differentiation was found at 2 dpi that identified significant clustering of ToCV and mock data sets when compared with naïve, suggesting an early response associated with the whitefly feeding. Interestingly, the ToCV-infected samples at 7 dpi and mocks at 7 and 14 dpi clustered tightly with the naive samples, indicating that infection with ToCV at 7 dpi and the effects of *Bemisia* feeding after 7 dpi did not alter the overall gene expression (Figure 1A).

We focused on the differentially expressed genes (DEGs) between mock and infected plants. The number of DEGs was 2009 (1163 up- and 936 down-regulated), 561 (246 up- and 315 down-regulated), and 5937 (2845 up- and 3092 down-regulated) at 2, 7, and 14 dpi, respectively (Figure 1B). The largest number of deregulated genes was at 14 dpi, whereas the lowest was found at 7 dpi, in agreement with the data distribution in the PCA, where the ToCV samples appeared close to the mock and naïve treatment. This indicates that while ToCV had a slight influence on the gene expression of the plant at the early stages of the infection, the effect of the virus became stronger at 14 dpi. Interestingly, at this later time point, over 80% of deregulated genes were specific and not shared with those observed at 2 and 7 dpi (Figure 1C). This finding suggests that these unique genes likely play a significant role in the development of virus symptoms.

### 3.2. Gene Ontology Enrichment Analysis in Response to ToCV

**Figure 2:**
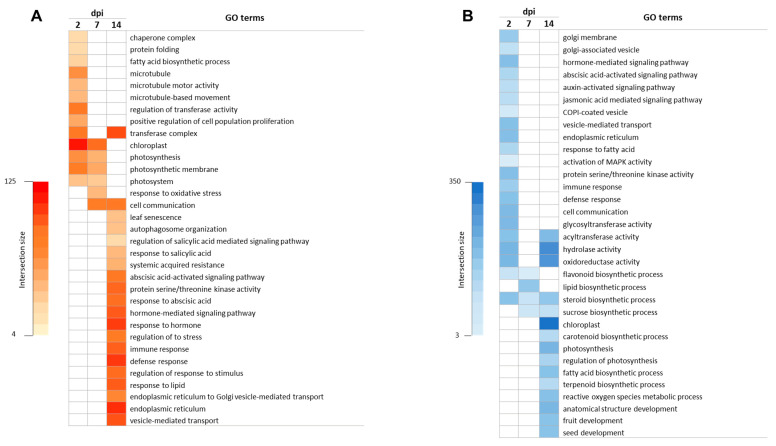
Gene ontology (GO) enrichment analysis of the differentially expressed genes (DEGs) in response to ToCV infection at 2, 7, and 14 days post inoculation (dpi). Enrichment of GO terms among the up-regulated (**A**) and down-regulated (**B**) DEGs. Each cell is colored based on the number of genes associated with the respective GO term.

To determine the functional roles of DEGs at each time point, we conducted GO enrichment analysis using the g:Profiler program [46]. A total of 454, 192, and 741 GO terms were significantly enriched at 2, 7, and 14 dpi, respectively, with a relatively higher number of enriched GO terms for up-regulated genes at 2 and 7 dpi and similar percentages for either up- or down-regulated genes at 14 dpi. The majority of the terms that were identified from up-regulated genes at 2 dpi were related to the microtubule and photosynthesis processes (Figure 2A), while some terms associated with defense and immune response, cell communication, and phytohormone signaling pathways were observed in down-regulated genes (Figure 2B). Out of the 192 terms significantly enriched at 7 dpi, the terms for up-regulated genes were mainly related to cell communication, photosynthesis, and response to stress (Figure 2A). In contrast, terms related to flavonoid biosynthesis, steroid metabolism, and chaperone activity were identified for down-regulated genes (Figure 2B). The number of enriched GO terms at 14 dpi was remarkably higher. Among the up-regulated DEGs, the most significant terms were related to cell communication, vesicular transport, and phytohormone signaling, with a particular emphasis on salicylic acid (Figure 2A). Regarding down-regulated genes, we discovered terms related to the response to photosynthesis, as well as the biosynthesis of lipids, carotenoids, flavonoids, and terpenoids (Figure 2B).

### 3.3. Impacted Pathways in ToCV Infection

**Figure 3:**
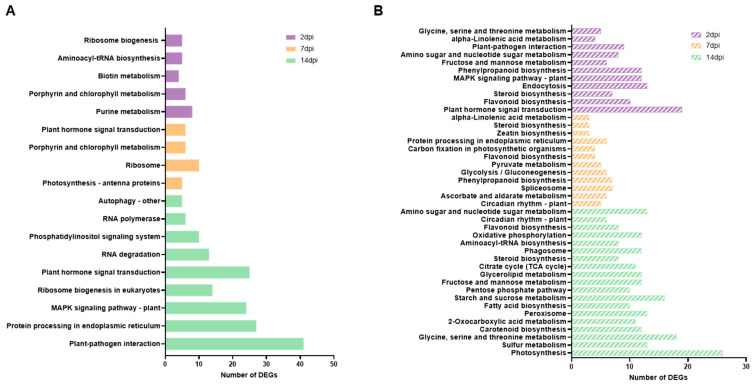
KEGG enrichment analysis. Top KEGG pathways enriched with up-regulated (**A**) and down-regulated (**B**) differentially expressed genes (DEGs) triggered by ToCV infection at 2, 7, and 14 days post-inoculation (dpi).

We also conducted a KEGG pathway analysis to assist in the identification of potential pathways, using the same software employed in the GO enrichment analysis. Our analysis revealed a greater number of KEGG pathways for down-regulated genes compared to up-regulated genes. Specifically, at 2 dpi, a total of 11 and 23 pathways were enriched for up-regulated and down-regulated genes, respectively. Notably, several important pathways such as plant hormone signal transduction, plant-pathogen interaction, and MAPK signaling were identified from the down-regulated genes at this time point (Figure 3A). Furthermore, we observed that some pathways associated with flavonoid biosynthesis and steroid biosynthesis were also repressed at 7 and 14 dpi (Figure 3B). The KEGG analysis performed at 7 dpi revealed 5 and 14 pathways related to up-regulated and down-regulated genes, respectively (Figure 3A,B). The most representative pathway categories identified from up-regulated genes included plant hormone signal transduction, ribosome, and photosynthesis (Figure 3A). Meanwhile, circadian rhythm, spliceosome, flavonoid biosynthesis, protein processing in the endoplasmic reticulum, and steroid biosynthesis-related pathways were identified among the down-regulated genes (Figure 3B). The analysis performed at 14 dpi showed that 15 pathways were enriched in up-regulated genes, while 49 pathways were identified in down-regulated genes. Among the top KEGG pathways from up-regulated genes were the MAPK signaling pathway, protein processing in the endoplasmic reticulum, plant hormone signal transduction, plant–pathogen interaction, and autophagy (Figure 3A). Conversely, down-regulated genes were significantly associated with pathways related to carotenoid biosynthesis, fatty acid biosynthesis, photosynthesis, and steroid biosynthesis (Figure 3B).

### 3.4. Characterizing Viral Small RNAs in ToCV-Infected Plants

**Figure 4:**
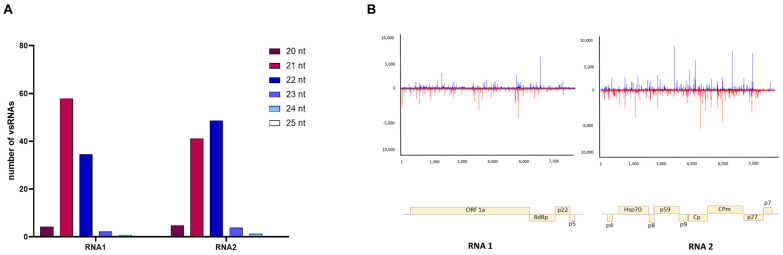
Global analysis of virus-derived small RNAs (vsRNAs) in tomato plants infected with ToCV. (**A**) Percentage of vsRNAs in the 20–25 nt reads pool mapped to RNA1 and RNA2 of the ToCV genome. (**B**) Single-nucleotide resolution maps of vsRNAs from tomato plants challenged by ToCV. Positive- and negative-strand reads are shown in blue and red, respectively. Genome organization of each viral genomic RNA is shown.

To gain insight into the siRNAs produced during ToCV infection, we conducted an analysis of vsRNA (20 to 25 nt) accumulation corresponding to ToCV genomic RNA1 and RNA2 using two pools of the same samples previously analyzed at 14 dpi in ToCV-infected plants for RNAseq. Our findings revealed that 235,638 and 1,011,948 siRNAs were mapped to viral RNA1 and RNA2, respectively. This indicates that the abundance of RNA2-specific vsRNAs was at least four times greater than that of RNA1-specific vsRNAs. Interestingly, the majority of sequenced vsRNAs originating from RNA1 were 21 nt in length, whereas those from RNA2 predominantly comprised 22 nt species (Figure 4A). This suggests that DCL4 played a prominent role in antiviral silencing for RNA1, whereas DCL2 was more actively involved in processing RNA2. Approximately equal ratios of vsRNAs were mapped to the positive and negative strands of each of the two viral genomic RNAs, respectively. Additionally, vsRNAs were generated from across the entire viral genome with several hot spots in regions encoding HSP70, probably interacting with virion tails during assembly and cell-to-cell movement, and the minor coat protein (CPm), linked to virion tails, plays roles in virus transmission by whitefly vectors, as well as in cell-to-cell movement and silencing suppression activity [1] (Figure 4B). Although there were slight variations in the relative abundance of vsRNAs targeting RNAs 1 and 2 between the duplicate libraries, no significant differences in the patterns described above were observed between the two replicates of each viral infection.

### 3.5. Enhanced ToCV Susceptibility in Tomato Plants after Silencing of Hsp90 and Sgt1 Genes

**Figure 5:**
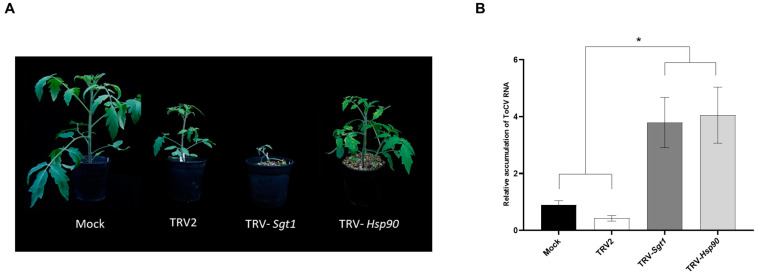
Impact of silencing of tomato *Hsp90* and *Sgt1* genes through virus-induced gene silencing on the susceptibility to ToCV infection. (**A**) Phenotypes observed in ToCV-infected tomato plants at 14 days post-inoculation (dpi) that were agroinfiltrated 7 days earlier with tobacco rattle virus (TRV) vector alone (TRV2), with the TRV Sgt1- and Hsp90-silencing constructs (TRV-*Sgt1* and TRV-*Hsp90*) and mock-inoculated (Mock). (**B**) Relative accumulation of ToCV RNA at 12 dpi analyzed by reverse transcription–quantitative polymerase chain reaction (RT-qPCR). The plants were agroinfiltrated 7 days earlier with TRV2, TRV-*Sgt1*, TRV-*Hsp90,* or Mock. Values were normalized using tomato elongation factor 1-α and Sand as reference genes, with Mock serving as the calibrator. Error bars represent standard errors of five biological replicates and an asterisk indicates a significant difference according to one-way ANOVA with *p* < 0.05.

The KEGG pathway enrichment analysis with up-regulated DEGs at 14 dpi showed that the plant–pathogen interaction KEGG pathway was the most significantly enriched in response to ToCV infection at 14 dpi (Figure 3A). Among the up-regulated DEGs included in this pathway, we focused on a chaperone *Hsp90* and the co-chaperone *Sgt1*, both involved in plant immunity, although their role in the tomato–ToCV interaction is unknown. In this context, a matter of interest was to investigate whether the silencing of this *Hsp90* and Stg1 might exert an influence on the accumulation of viral RNA in plants. Initial infection of tomato plants with TRV-*Sgt1* or TRV-*Hsp90* was followed by a challenge-inoculation with ToCV after 7 days. Silencing of *Sgt1* led to plant lethality by 21 dpi, while *Hsp90* silencing induced only a comparatively mild phenotypic response (Figure 5A). RT-qPCR analysis at 7 dpi showed that the levels of target mRNAs were reduced in the silenced plants compared to the control plants inoculated with the empty TRV vector (Figure S1A,B). These plants were inoculated using ToCV-viruliferous whiteflies, and viral RNA accumulation was assessed 12 days after whitefly-mediated inoculation in *Hsp90* and *Sgt1*-silenced plants to prevent the development of the severe phenotype for *Sgt1*. The results showed a notable increase in the accumulation of ToCV RNA in the silenced plants, with levels approximately four times higher than those in the control TRV-infected plants (Figure 5B). Moreover, no significant alterations were observed in the TRV accumulation levels (Figure S1C). These findings suggest that *Hsp90* and *Stg1* might play an important role in basal resistance against ToCV.

## 4. Discussion

In our study, our objective was to provide a comprehensive insight into the alterations within the tomato transcriptome throughout the course of ToCV infection. We accomplished this by analyzing the dynamic transcriptional responses of tomato plants at different time points following their infection by *B. tabaci*. Our findings revealed that gene expression in plants undergoes significant changes over time. We observed a large number of DEGs at 2 dpi (2009), with a substantial decrease at 7 dpi (561). However, at 14 dpi, we observed a remarkable reprogramming of the plant transcript profile, with 5937 genes exhibiting differential expression (Figure 1B). These findings highlight the dynamic nature of the plant transcriptome during the progression of ToCV infection and offer a snapshot of a particular stage in the plant’s life cycle and the course of infection. On the other hand, the PCA analysis indicated that mock and ToCV samples formed a distinct cluster from naïve samples at 2 dpi (Figure 1A), suggesting that the host can sense the whitefly infestation regardless of whether the whitefly was viruliferous or non-viruliferous. However, the significant deregulation of 2009 genes observed at 2 dpi in the ToCV samples, compared to the mock samples, strongly suggests that ToCV may have significant effects on the whitefly–plant interaction. Interestingly, the pathways related to flavonoids [54,55] and the steroid biosynthesis involved in resistance to insect herbivores were repressed during ToCV infection (Figure 3B). Yao et al. (2019) [55] demonstrated that tomatoes with high flavonoid levels exhibited resistance to *B. tabaci*, resulting in a decrease in both the primary and secondary spread of TYLCV. Furthermore, steroid plant hormones such as Brassinosteroids (BRs), which are involved in plant growth and development [56], also play a role in plant–herbivore interactions, likely by regulating glucosinolate biosynthesis [57,58,59,60]. Additionally, several studies have suggested that BRs also function in plant immunity by inducing plant defenses against viruses [61,62,63]. Further studies are required to gain a deeper understanding of the specific mechanisms underlying the repression of flavonoids and BRs during ToCV infection and to investigate how this repression may influence insect resistance and the spread of the virus.

The GO analysis revealed there was an up-regulation of genes associated with the microtubule-based process at 2 dpi (Figure 2A), a phenomenon frequently observed in response to viral infections [64,65,66]. Microtubules are known to play crucial roles in various biological processes, such as virus movement, the assembly of viral replication complexes, and the formation of transmission bodies that facilitate virus transmission between plants via insect vectors [67,68]. However, the rationale behind their up-regulation in ToCV-infected plants remains unknown, and further investigation will be necessary to uncover any underlying causes. GO annotation also revealed an enrichment of genes related to photosynthesis activity and chloroplast organization and structure at 2 and 7 dpi. However, an intriguing contrast emerged, as these same GO terms exhibited down-regulation at 14 dpi (Figure 2 and Figure 3). This underscores once again the need to conduct transcriptomic analyses at different time points during viral infection. Considering that chloroplasts function as factories for the synthesis of key signaling molecules such as salicylic acid (SA) and jasmonic acid (JA) for host defense responses against viruses [69], it is reasonable to speculate that the activation of these pathways constitutes a direct reaction to viral infection. On the contrary, at 14 dpi, we observed a down-regulation of genes linked to chloroplasts and photosynthesis, consistent with previous findings reported by Çevik et al., 2021 [70]. This down-regulation was accompanied by an up-regulation of genes involved in the SA signaling pathway. Such patterns are frequently documented in virus-infected tissues and are believed to underlie the development of chlorosis symptoms commonly associated with viral infections [9,71]. Indeed, several genes related to leaf senescence and autophagy activity [72,73] were up-regulated during ToCV infection at 14 dpi (Figure 4A). Similar findings were reported by [17] in ToCV-infected plants, despite differences in the experimental conditions, tomato cultivars, and virus isolates used in their study. These findings suggest that diverse genes related to the defense pathways of tomato plants are expressed during the late stages of ToCV infection [74,75].

We found that RNA2 spawns much more abundant vsRNAs than RNA1, which reflects the higher replication rate of the RNA2 potentially leading to the production of more abundant dsRNA replication intermediates for dicing. The differential vsRNA length distribution patterns between RNA1 and RNA2 raise intriguing questions about the underlying molecular mechanisms governing vsRNA biogenesis in ToCV. The observed preference for 21 nt vsRNAs from RNA1 and 22 nt vsRNAs from RNA2 may reflect distinct mechanisms to suppress gene silencing by ToCV viral suppressors of RNA silencing (VSRs), which include the p22 protein encoded by RNA1, as well as the coat protein (CP) and CPm encoded by RNA2 [76]. Moreover, it is plausible that these VSRs exhibit an asynchronous temporal expression pattern, given that the replication of RNA2 is delayed compared to that of RNA1 [77], contributing to the observed profile of vsRNA. It has been well established that DCL4 functions as the primary sensor of viral dsRNAs, producing vsRNAs 21 nt in length [23]. However, consistent with a previous report [35], we found that vsRNAs originating from ToCV were predominantly 22 nt in length, likely orchestrated by tomato orthologs of DCL2. Considering that VSRs have the potential to interfere with host factors involved in antiviral silencing [78], the prevalence of 22 nt vsRNAs may be a consequence of the ability of ToCV-encoded VSRs [76] to disrupt the functioning of DCL4. Similar mechanisms have been observed in other viruses, such as the turnip crinkle virus, where the VSR CP inhibits DCL4 activity, and consequently, DCL2 becomes the major contributor to vsRNA biogenesis [24]. Interestingly, our transcriptome analysis revealed that single infections with ToCV led to a significant up-regulation in the expression levels of DCL2b (2.70-fold), DCL2d (9.10-fold), and DCL4 (1.67-fold) when compared to mock plants (Table S1). Furthermore, Wang et al. (2018) [79] documented that tomato DCL2b ranks among the most abundantly expressed members of the DCL2 family and offers enhanced protection against tobacco mosaic virus. Alternatively, ToCV could target the amplification step of the RNA silencing that promotes the transformation of targeted ssRNA into dsRNA by RDR6, thus inhibiting the production of 21 nt secondary siRNAs [80]. Approximately equal amounts of vsRNAs were mapped to the positive and negative strands of RNA1 and RNA2 viral genome of ToCV, respectively (Figure 4B), indicating vsRNAs were likely produced from double-stranded replicative intermediates.

The *Hsp90*–*Sgt1* complex plays a critical role in regulating the plant immune system against pathogens, including plant viruses [81]. In this study, we observed an up-regulation of *Hsp90* (1.47-fold) and *Sgt1* (8.35-fold) expression at 14 dpi following ToCV infection (Table S1). Notably, the silencing of these genes resulted in a higher level of ToCV accumulation in tomato plants (Figure 5B), suggesting their potential involvement in antiviral responses specific to the tomato–ToCV pathosystem. Additionally, silencing of *Sgt1* through VIGS led to cell death, whereas silencing of the *Hsp90* did not cause noticeable differences when compared to control TRV-infected plants. Our results are in line with those of [82], who demonstrated that silencing the tomato *Hsp90* and *Sgt1* genes resulted in increased TYLCV accumulation. However, while they reported that silencing of both genes led to cell death, we observed this phenotype only in *Sgt1* tomato plants (Figure 5A). Furthermore, we did not observe any relief of the phenotype caused by ToCV infection in the *Sgt1*-silenced plants, in contrast to their observations in plants infected with TYLCV [82]. This difference could be attributed to the specific silencing of distinct *Hsp90* genes in each study (our investigation focused on Solyc07g047790, whereas their study emphasized Solyc12g015880). Interestingly, it has been reported that the infection of various RNA viruses, such as tomato spotted wilt virus and potato virus X, is impaired in *Sgt1*-silenced *N. benthamiana*, despite the fact that these viral infections strongly upregulate *Sgt1* expression in the plant [83,84]. Additionally, the interaction between *Hsp90* and the bamboo mosaic virus 3′UTR is implicated in promoting viral accumulation, likely aiding the entry of viral replication complexes into chloroplasts [85]. Therefore, the *Hsp90*–*Sgt1* complex appears to serve dual functions, playing a crucial role in basal resistance against certain viruses while also potentially acting as a proviral factor for others.

We believe that our study enhances our comprehension of the molecular responses occurring in ToCV-infected tomato plants. This knowledge may aid in the identification of potential genes implicated in defensive responses, making them good candidates for future breeding efforts. These candidates could serve as targets for the development of new strategies aimed at controlling the disease through the modulation of endogenous pathways.

## Data Availability

The data for this study have been deposited in the European Nucleotide Archive (ENA) at EMBL-EBI under accession number PRJEB67704 (https://www.ebi.ac.uk/ena/browser/view/PRJEB67704, accessed on 23 October 2023).

## Supplementary Materials

The following supporting information can be downloaded at: https://www.mdpi.com/article/10.3390/v15122370/s1, Figure S1: Expression analysis of targeted *Sgt1* and *Hsp90* genes in tomato plants subjected to virus-induced gene silencing using tobacco rattle virus (TRV) vector. Table S1: Calculated fold-changes of expression of target genes *Hsp90*, *Sgt1*, *DCL2b*, *DCL2d* and *DCL4*.
