# Supplementary material for: Enhanced Susceptibility to Tomato Chlorosis Virus (ToCV) in Hsp90- and Sgt1-Silenced Plants: Insights from Gene Expression Dynamics
Source: Viruses. 2023 Nov 30;15(12):2370. doi: 10.3390/v15122370 (PMC10747942; doi:10.3390/v15122370)
Supplement: Supplementary file 1 [file viruses-15-02370-s001.zip › viruses-2721142-supplementary.pdf]

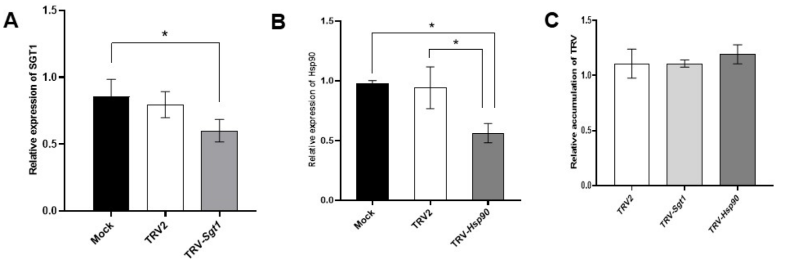

Figure S1. Expression analysis of targeted genes Sgt1 (A) and Hsp90 (B) in tomato plants subjected to virus-induced gene silencing using Tobacco rattle virus (TRV) vector. (A) Relative expression levels of Sgt1 analyzed by Reverse Transcription-quantitative Polymerase Chain Reaction (RT-qPCR) in tomato plants infected with TRV vector alone (TRV2), TRV carrying Sgt1 silencing constructs (TRV-Sgt1), and mock-inoculated (Mock). Transcript levels were normalized using tomato genes encoding elongation factor 1-α and SAND as internal references, with Mock as the calibrator. (B) Relative expression levels of Hsp90 analyzed by RT-qPCR in tomato plants infected with TRV2, TRV-Hsp90, and Mock. Gene expression levels were normalized using the same reference genes as in (A). (C) Quantification of the relative accumulation of TRV RNA in plants infected with TRV2 and in silenced plants (TRV-Sgt1 and TRV-Hsp90). The expression levels were normalized using tomato genes for elongation factor 1-α and SAND, with TRV2 as the calibrator. All samples were collected at 19 dpi to anticipate the observed decline. Error bars represent standard errors of five biological replicates, and an asterisk indicates a significant difference, as determined by one-way ANOVA with $P < 0.05$

Table S1. Calculated fold-changes of expression of target genes *Hsp90*, *Sgt1*, *DCL2b*, *DCL2d* and *DCL4*.

Gene ID	Gene name	ToCV/Mock (Log_2FC)[1]	ToCV/Mock (FC)[1]
Solyc07g047790	*Hsp90*	0.53318	1.47
Solyc06g0364201	*Sgt1*	3.06260	8.35
Solyc11g008540	*DCL2b*	1.43458	2.70
Solyc11g008530	*DCL2d*	3.20032	9.10
Solyc07g005030	*DCL4*	0.74274	1.67

[1] FC Fold Change.
